# Promoter methylation of *ADAMTS1* and *BNC1* as potential biomarkers for early detection of pancreatic cancer in blood

**DOI:** 10.1186/s13148-019-0650-0

**Published:** 2019-04-05

**Authors:** Maryam A. L. Eissa, Lane Lerner, Eihab Abdelfatah, Nakul Shankar, Joseph K. Canner, Nesrin M. Hasan, Vesal Yaghoobi, Barry Huang, Zachary Kerner, Felipe Takaesu, Christopher Wolfgang, Ruby Kwak, Michael Ruiz, Matthew Tam, Thomas R. Pisanic, Christine A. Iacobuzio-Donahue, Ralph H. Hruban, Jin He, Tza-Huei Wang, Laura D. Wood, Anup Sharma, Nita Ahuja

**Affiliations:** 1 Department of Surgery, The Johns Hopkins University School of Medicine, Baltimore, MD USA; 2 Department of Oncology, The Sidney Kimmel Comprehensive Cancer Center, The Johns Hopkins University School of Medicine, Baltimore, MD USA; 3 Johns Hopkins Institute for NanoBioTechnology, The Johns Hopkins University, Baltimore, MD USA; 4 Department of Pathology, Memorial Sloan Kettering Cancer Center, New York, USA; 5 Department of Pathology, The Johns Hopkins University School of Medicine, Baltimore, MD USA; 6 The Sol Goldman Pancreatic Cancer Research Center, The Sidney Kimmel Comprehensive Cancer Center, Johns Hopkins Hospital, Baltimore, MD USA; 7 Department of Surgery, Yale-New Haven Health, Yale University, School of Medicine, P.O. Box 208062, New Haven, CT 06520-8062 USA

**Keywords:** Cell-free DNA, MOB, *ADAMTS1*, *BNC1*, Methylation, Biomarker, Pancreatic cancer

## Abstract

**Background:**

Despite improvements in cancer management, most pancreatic cancers are still diagnosed at an advanced stage. We have recently identified promoter DNA methylation of the genes *ADAMTS1* and *BNC1* as potential blood biomarkers of pancreas cancer. In this study, we validate this biomarker panel in peripheral cell-free tumor DNA of patients with pancreatic cancer.

**Results:**

Sensitivity and specificity for each gene are as follows: *ADAMTS1* 87.2% and 95.8% (AUC = 0.91; 95% CI 0.71–0.86) and *BNC1* 64.1% and 93.7% (AUC = 0.79; 95% CI 0.63–0.78). When using methylation of either gene as a combination panel, sensitivity increases to 97.3% and specificity to 91.6% (AUC = 0.95; 95% CI 0.77–0.90). Adding pre-operative CA 19-9 values to the combined two-gene methylation panel did not improve sensitivity. Methylation of *ADAMTS1* was found to be positive in 87.5% (7/8) of stage I, 77.8% (7/9) of stage IIA, and 90% (18/20) of stage IIB disease. Similarly, *BNC1* was positive in 62.5% (5/8) of stage I patients, 55.6% (5/9) of stage IIA, and 65% (13/20) of patients with stage IIB disease. The two-gene panel (*ADAMTS1* and/or *BNC1*) was positive in 100% (8/8) of stage I, 88.9% (8/9) of stage IIA, and 100% (20/20) of stage IIB disease. The sensitivity and specificity of the two-gene panel for localized pancreatic cancer (stages I and II), where the cancer is eligible for surgical resection with curative potential, was 94.8% and 91.6% respectively. Additionally, the two-gene panel exhibited an AUC of 0.95 (95% CI 0.90–0.98) compared to 57.1% for CA 19-9 alone.

**Conclusion:**

The methylation status of *ADAMTS1* and *BNC1* in cfDNA shows promise for detecting pancreatic cancer during the early stages when curative resection of the tumor is still possible. This minimally invasive blood-based biomarker panel could be used as a promising tool for diagnosis and screening in a select subset of high-risk populations.

**Electronic supplementary material:**

The online version of this article (10.1186/s13148-019-0650-0) contains supplementary material, which is available to authorized users.

## Introduction

Pancreatic cancer is the third leading cause of both domestic and global cancer-related mortality [1]. It has a poor prognosis with an overall 5-year survival rate of 7% [2, 3], as the cancer often grows insidiously and initially does not cause symptoms. The poor prognosis is at least in part due to the absence of specific symptoms early in the course of the disease and lack of effective diagnostic methods. Over 75% of pancreatic cancer cases are diagnosed at stage III/IV [4]. Yet, surgical resection is currently the only potentially curable therapy [5, 6]. Therefore, there is an urgent need for reliable, non-invasive, and cost-effective early detection method for biomarkers of pancreatic cancer.

DNA methylation plays an important role in cancer development and progression [7]. DNA methylation can alter the DNA chromatin structure and can lead to the silencing of tumor suppressor genes or activation of oncogenes [7, 8]. Many of these changes occur early in tumorigenesis, making epigenetic modifications a promising target as biomarkers for the early detection of cancer [9–12]. DNA methylation changes occur early in cancer formation for many cancer types including colorectal [13], breast [14], and pancreas [10–12, 15] and that methylation changes can be potent biomarkers for early detection [16–18]. The recent FDA approval of methylation biomarkers for colorectal cancer (Cologuard®, Epi proColon®) as well as bladder cancer (Exact Sciences *Inc.*) serves as a blueprint for taking these biomarkers to patient care. In pancreatic cancer, CA19-9, a carcinoembryonic antigen, is approved by the FDA for prognostic surveillance of known pancreatic cancer patients; however, it is considered to have low sensitivity and specificity for pancreatic cancer detection [19–22]. Currently approved tests for the early detection of pancreatic cancer do not exist.

Cell-free DNA (cfDNA) from tumor tissues has been found within plasma samples from cancer patients’ peripheral circulating blood. Additionally, the amount of total cfDNA in cancer patients is higher than in normal populations [23–27]. This cfDNA is composed of short segments of nucleic acids that are not associated with cells or cell fragments. Importantly, cfDNA reflects the genetic and epigenetic makeup of the tumor from which it originates, making it a desirable and highly specific biomarker for the early detection of cancer. Tests for circulating tumor DNA can be improved when they are combined with highly selected protein markers [26].

We have been investigating the role of epigenetic changes in pancreatic cancer over the past few years and have previously published on the widespread methylation changes that occur in pancreatic cancer. Our studies identified two biomarkers *ADAMTS1* (A disintegrin and metalloproteinase with thrombospondin motifs 1) and *BNC1* (zinc finger protein basonuclin-1) as highly sensitive markers for the early detection of pancreatic cancer in tissue [15]. In a previous study of selected pancreatic cancer patients, we reported promising sensitivity and specificity with the two genes at 81% and 85% respectively using non-invasive fluids [15].

In this study, we took advantage of a newly developed [28, 29] highly reliable technique called methylation on beads (MOB). DNA methylation of genes *ADAMTS1* and *BNC1* was studied using cfDNA in a large cohort of patients with varying stages of pancreatic cancer and an age-matched normal group to determine the sensitivity, specificity, and applicability of this two-gene panel as a non-invasive biomarker set for the early detection of pancreatic cancer. Furthermore, pre-operative CA 19-9 levels were also compiled to study the significance of their values, either alone or in combination with the methylation-based gene panel, in detecting early stage pancreatic cancer.

## Results

### Patient and control cohorts

Patients with different stages of pancreatic cancer undergoing surgical resection were enrolled in this study. 20.5% (8/39) were found to have stage I cancer, 23.1% (9/39) stage IIA, 51.3% (20/39) stage IIB, and only 5.1% (2/39) stage III/IV cancers. Patients with stage III/IV cancers were surgically explored and the surgery aborted due to the advanced stage of the disease. There was no significant age difference between the cancer and control groups (mean age 60.1 years vs. 65.5 years) (Table 1). Cigarette smoking, pancreatitis, and diabetes mellitus have all been identified as important risk factors for pancreatic cancer in previous studies [30–32]. Our study recruited population-based matched controls to determine the specificity of our markers. Of note, amongst the controls, 48% had diabetes mellitus, 38% had hypertension, and 17% were current smokers. Patients with pancreatitis were undergoing surgery for pain control that was unresponsive to long-standing medical therapy. Patients with pancreatitis had median 84 months of disease prior to undergoing surgery. There was a significant racial difference between the two cohorts used in the study (76% of control group were African-American vs 5% of cancer cases; *p* < 0.05) (Table 1). The majority of the cancer patients (69.2%) were treated with pancreaticoduodenectomy, while 10.3% and 15.4% had total and distal pancreatectomy respectively, and in 5.1%, the resection was aborted (Additional file 1: Table S1).

**Table 1:** Patient demographics, tumor clinicopathologic features

	Control (*n* = 95)	Cancer (*n* = 39)	*p* value (cancer vs control)	Pancreatitis (*n* = 8)
Average age, years (range)	65.5 (21–96)	60.1 (29–83)	0.059	46.6 (29–70)
Gender				
Male (%)	41 (43%)	26 (67%)	0.013	4 (50%)
Female (%)	54 (57%)	13 (33%)		4 (50%)
Race				
White (%)	22 (23%)	34 (87%)		6 (78%)
Black (%)	72 (76%)	2 (5%)	< 0.05	1 (11%)
Others (%)	0 (0%)	3 (8%)		1 (11%)
Not specified (%)	1 (1%)	0 (0%)		0 (0%)
CA 19-9 level*	–	*n* = 34	N/A	*n* = 1
Average	–	665.3		–
Median	–	47.6		–
Highest	–	12,302.5		–
Lowest	–	1		16.1
Smoking status	*n* = 95	*n* = 39		*n* = 8
Never	38 (40%)	22 (56%)		5 (63%)
Former	40 (42%)	10 (26%)	0.159	3 (38%)
Current	16 (17%)	7 (18%)		0 (0%)
Not specified	1 (1%)	0 (0%)		0 (0%)
Average tumor size (range, cm)**	–	3.2 (0.9–8)	N/A	–
Differentiation		*n* = 39	N/A	
Well	–	1 (3%)		–
Moderate	–	17 (44%)		–
Poor	–	12 (31%)		–
Undifferentiated	–	2 (5%)		–
Not specified	–	7 (18%)		–

*CA 19-9: five values unavailable for PDAC patient and four unavailable for pancreatitis

**Tumor size: three PDAC, no resection done, N/A: Not applicable

### *ADAMTS1* and *BNC1* methylation status in pancreatic cancer

**Fig. 1:**
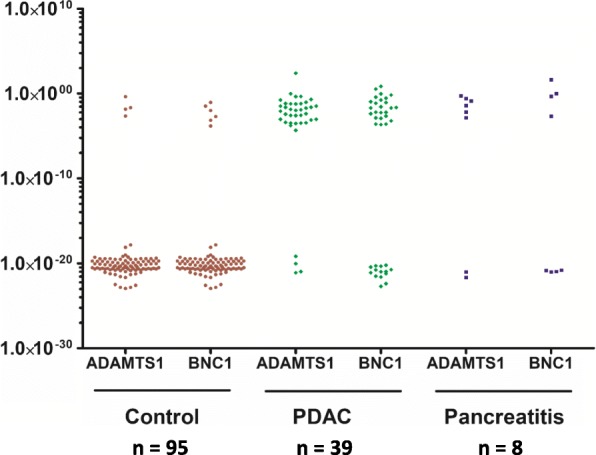
Methylation of *ADAMTS1* and *BNC1* genes in control and PDAC cases

cfDNA isolated from the blood samples obtained from pancreatic cancer patients (*n* = 39), pancreatitis (*n* = 8), and control group (*n* = 95) was evaluated (Fig. 1). Overall methylation of either gene (*ADAMTS1* and/or *BNC1*) was observed in 97.4% of cancer patients (38/39) compared to 8.4% of control patients (8/95). Methylation of *ADAMTS1* was detected in 87.2% (34/39) pancreatic adenocarcinomas. *BNC1* methylation was detected in 65.1% (25/39) of cases. Methylation analysis was also performed in a series of non-cancer individuals (*n* = 95). Amongst this group, methylation of *ADAMTS1* was detected in 4.2% (4/95), while *BNC1* was detected in 6.3% (6/95) of cases. 97.4% (38/39) and 8.4% (8/95) of patients and controls respectively showed evidence of methylation of either of the two genes. Among controls, 17% and 42% of patients were current and former smokers respectively, while 55.4% were type II diabetic. The use of the two-gene combination (*ADAMTS1* and/or *BNC1*) panel made a demonstrable difference of the predictive power of both the genes as compared to the use of a single gene by itself (Table 2). Using the presence of methylation in either gene, pancreatic cancer was detected in 97.4% (38/39) of all pancreatic cancer patients.

**Table 2:** Methylation frequency of two-gene panel (*ADAMTS1* and/or *BNC1)* vs frequency of CA 19-9 elevation in control, PDAC, and pancreatitis

Patients (*n*)	Control		PDAC		Pancreatitis	
	Positive/total	Positive (%)	Positive/total	Positive (%)	Positive/total	Positive (%)
*ADAMTS1*	4/95	4.2	34/39	87.2	6/8	75
*BNC1*	6/95	6.3	25/39	65.1	4/8	50
*ADAMTS1* and *BNC1*	2/95	2.1	21/39	53.8	3/8	37.5
*ADAMTS1 and/or BNC1*	8/95	8.0	38/39	97.4	7/8	87.5
CA 19-9 (*n* = 34)	N/A		19/34	55.9	N/A	
*ADAMTS1* and *BNC1* and CA 19-9*	N/A		6/34	17.6	N/A	
*ADAMTS1* and/or *BNC1* + CA 19-9	N/A		38/39	97.4	N/A	

*Positive for all three, N/A: Not applicable

We also investigated the utility of CA19-9 as an early detection biomarker. CA19-9 values were available in 87.2% (34/39) pancreatic cancer patients. Amongst these patients, CA19-9 was elevated above the normal threshold (> 36 units) in 55.9% (19/34) of pancreatic adenocarcinoma group. Thus, CA19-9 failed to detect the presence of pancreatic cancer in 44.1% (15/34) of the cancer cases. We next tested the utility of CA19-9 as an additive biomarker. The combined sensitivity of *ADAMTS1* and CA 19-9 was 87.2% compared to 87.2% for *ADAMTS1* alone while that of *BNC1* with CA 19-9 was 89.2% compared to 64.1% with *BNC1* alone. However, there was no improvement in sensitivity on the addition of CA 19-9 to the two-gene methylation panel (*ADAMTS1* and/or *BNC1* +/− CA19-9, 97.4% both) (Table 2).

### *ADAMTS1* and *BNC1* methylation status in various stages of cancer

Methylation of *ADAMTS1* was found to be positive in cfDNA of 87.5% (7/8) patients with stage I cancer, 77.8% (7/9) of stage IIA, 90% (18/20) of stage IIB, and 100% (2/2) of stage III/IV pancreatic cancer patients. Similarly, *BNC1* was positive in 62.5% (5/8) of stage I patients, 55.6% (5/9) of stage IIA, 65% (13/20) of stage IIB, and 100% (2/2) of stage III/IV pancreatic cancers. When considering the combination panel of both genes (*ADAMTS1* and/or *BNC1*), the combined panel was positive in 100% (8/8) of stage I, 88.9% (8/9) of stage IIA, 100% (20/20) of stage IIB, and 100% (2/2) of stages III and IV pancreatic cancers (Table 3). The difference in positive methylation prevalence between different stages was not statistically significant. When looking at CA19-9 by different cancer stages, CA19-9 was positive in only 57.1% (4/7) of stage I patients, 44.4% (4/9) of stage IIA, 64.7% (11/17) of stage IIB, and 0% (2/2) of stage III/IV pancreatic cancers. The combined two-gene panel of *ADAMTS1* and/or *BNC1* detected 94.8% (37/39) of all early-stage PDAC patients (stages I and II) who are eligible for surgical curative resection vs 57.6% for CA19-9 (19/33) alone (*p* = 0.0003).

**Table 3:** Methylation frequency of two-gene panel (*ADAMTS1* and/or *BNC1*) vs frequency of CA 19-9 elevation in different stages of pancreatic ductal adenocarcinoma

Patients	Stage I (%)		Stage IIA (%)		Stage IIB (%)		Stages III–IV (%)	
	Positive/total	Positive (%)	Positive/total	Positive (%)	Positive/total	Positive (%)	Positive/total	Positive (%)
*ADAMTS1*	7/8	87.5	7/9	77.8	18/20	90	2/2	100
*BNC1*	5/8	62.5	5/9	55.6	13/20	65	2/2	100
*ADAMTS1* and/or *BNC1*	8/8	100	8/9	88.9	20/20	100	2/2	100
CA 19-9 (*n* = 34)*	4/7	57.1	4/9	44.4	11/17	64.7	0/1	0
*ADAMTS1* and/or *BNC1* + CA 19-9	8/8	100	8/9	88.9	20/20	100	2/2	100

*Missing two CA 19-9 values, so % out of total recorded CA 19-9 values for each stage

### Evaluation of the two-gene panel in pancreatitis

Patients with pancreatitis have a higher risk of developing PDAC; therefore, cfDNA samples from eight patients with chronic pancreatitis were also evaluated. Using the combination panel (*ADAMTS1* and/or *BNC1*), 87.5% (7/8) of chronic pancreatitis patients had positive methylation (Table 2).

### Sensitivity, specificity, and the accuracy of the biomarker

**Fig. 2:**
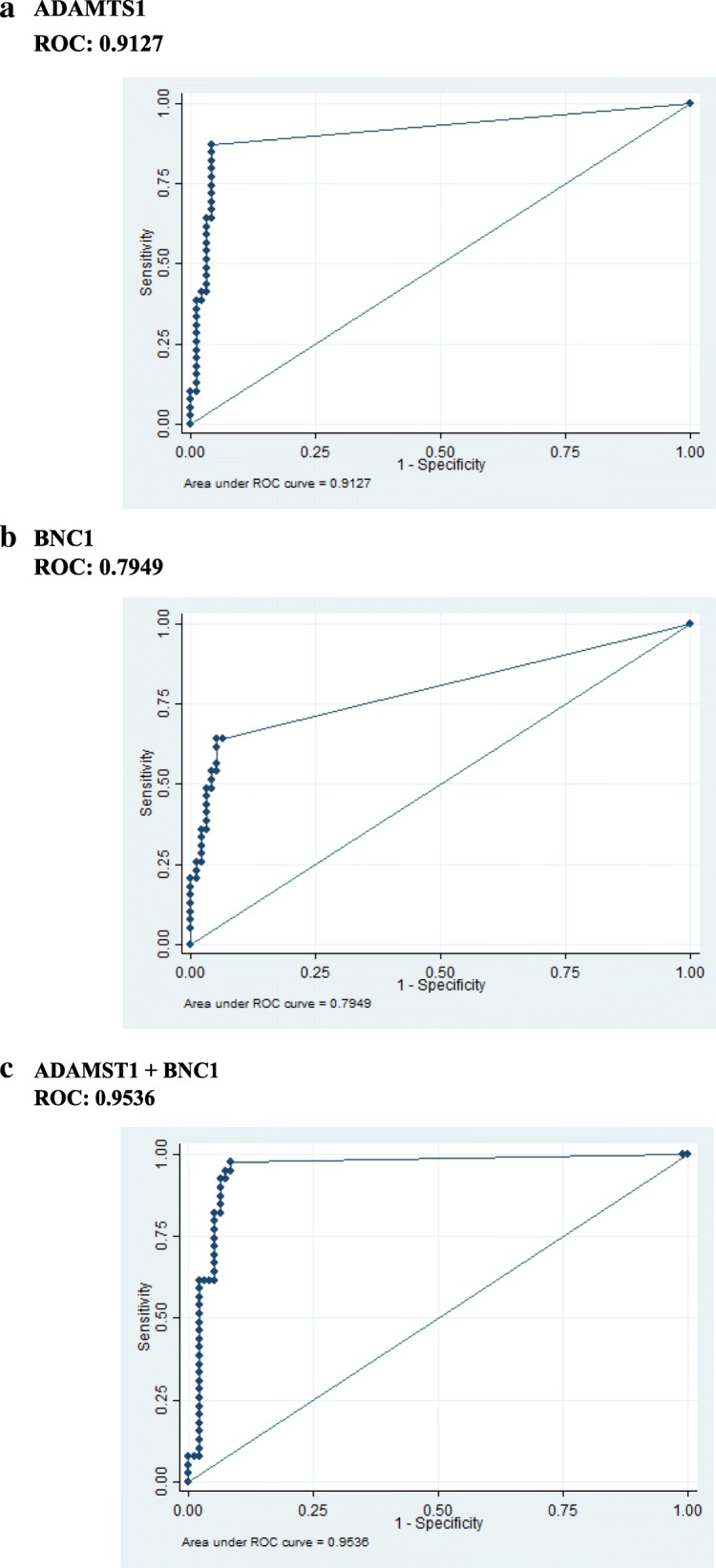
Sensitivity and specificity of both genes. ROC curves for various genes. (**a**, **b**) ROC curves are represented for individual genes (*ADAMTS1* and *BNC1*) and **c** combined methylation status of the genes (*ADAMTS1* + *BNC1*) from the plasma samples

We calculated a methylation positivity for each gene independently and as a combination panel. There was no significant difference in methylation level between different races, genders, or age groups. We then used these values to generate a receiver operating characteristic curve (ROC) to determine the sensitivity and specificity for each gene at the optimal cutoff value. The sensitivity and specificity of *ADAMTS1* was 87.2% and 95.8%, respectively. *BNC1* demonstrated a sensitivity and specificity of 64.1% and 93.7%, respectively. The combination panel of methylation of either gene demonstrated a sensitivity and specificity of 97.4% and 91.6%, respectively. The area under the curve (AUC) for *ADAMTS1* was 0.91 (95% CI 0.85–0.95), while AUC of *BNC1* was 0.79 (95% CI 0.70–0.85). The AUC of the two-gene combination panel was 0.95 (95% CI 0.91–0.98) (Table 4) (Fig. 2a–c). The AUC for early stage, resectable pancreatic cancers was 0.91 (95% CI 0.85–0.95) for *ADAMTS1*, AUC 0.78 (95% CI 0.70–0.85) for *BNC1* alone, while the AUC of the two-gene combination was 0.95 (95% CI 0.90–0.98).

**Table 4:** Gene methylation detection in plasma samples for cancer and controls

	Sensitivity	Specificity	PPV	NPV	AUC
CA19-9	55.9% (19/34)	NA	NA	NA	NA
*ADAMTS1*	87.2% (34/39)	95.8% (91/95)	89/5% (34/38)	95.8% (91/96)	0.91
*BNC1*	64.1% (25/39)	93.7% (89/95)	80.6% (25/31)	86.4% (89/103)	0.79
*ADAMTS1* and/or *BNC1*	97.4% (38/39)	91.6% (87/95)	82.6% (38/46)	98.9% (87/88)	0.95
*ADAMTS1* and/or *BNC1* and/or CA19-9	97.4% (38/39)	NA	NA	NA	NA
*ADAMTS1* + CA19-9	87.2% (34/39)	NA	NA	NA	NA
*BNC1* + CA19-9	89.2% (33/37)	NA	NA	NA	NA

N/A: Not applicable

## Discussion

DNA methylation plays an important role in the epigenetic modification of the cells. Hypermethylation of CpG islands in promoter regions of tumor suppressor genes can lead to gene downregulation, gene silencing, or aberrant post-translational modifications, all of which can contribute to cancer development. Identification of reliable early detection markers to identify cancers in a non-invasive fashion using DNA methylation markers has shown exciting promise.

An array of noninvasive clinical tests has been developed for colorectal (CRC) cancer screening, including the fecal occult blood test, the fecal immunochemical test, the fecal-based DNA test, and the blood-based DNA test (the SEPT9 assay). Another popular commercially available test is a non-invasive colon cancer screening test based on DNA obtained from the stool, Cologuard® (Exact Sciences). This test takes a multi-target approach and screens for altered DNA, hemoglobin biomarkers, methylation of genes (*NDRG4* and *BMP3*), and *KRAS* point mutations for early detection of CRC [33]. Epi proColon® based on methylated septin 9 which is altered in colorectal cancer tumor cells more often than in normal cells is the first and only FDA-approved blood-based test the detection of colorectal cancer [34–36]. Another commercial test, Oncotype DX, based on a multigene approach, has been developed for the early detection of breast [37], colon [38], and prostate [39] cancers. However, given the fact that one gene rarely defines the status of tumor formation, a multi-gene approach has been used in order to develop a detection method that can recognize early events in tumorigenesis [40, 41].

Early detection of cancer is crucial to treatment. Early detection can mean less invasive or risky surgical procedures prior to metastasis, along with improved survival. This is exemplified by a diversity of public health statistics on cancer. For breast cancer, the 5-year survival of women with early stage diagnosis is as high as 90%, but as low as 15% with advanced disease [42]. Early detection through screening has increased the 5-year survival rate to 88% and 90% in lung and CRC respectively [43, 44]. Early detection of pancreatic cancer is also associated with better prognosis [45–47].

Earlier detection holds great promise for patients with pancreatic cancer [48]. Chemotherapy has only been shown to have moderate success, and surgical resection remains the only curative option but less than 20% of patients present with resectable tumor [46, 49, 50]. Additionally, the most important factor in determining whether a patient is a candidate for surgical intervention is the presence or absence of metastases. However, pancreatic cancer is often either discovered incidentally or remains asymptomatic for a long period, growing to become either locally advanced or metastatic before causing symptoms such as gastric pain, jaundice, or other signs of biliary obstruction. At that point, 80–85% of patients present with already advanced, unresectable disease. Developing an early diagnostic approach is one possible solution to increase detection at resectable stages of disease [25–27]. Most studies concerning the use of tumor markers in pancreatic cancer have been directed toward the use of serum-based testing. However, currently, no available serum-based marker can be used to aid in the diagnosis of pancreatic cancer because of suboptimal sensitivity and specificity. There is a dire need for an early detection test that exhibits a high sensitivity and specificity. To achieve this, the focus of early detection biomarkers should be to identify early stages (stages I and II) of the disease.

CA 19-9 is currently the only biomarker routinely utilized clinically in the management of pancreatic cancer, generally as a method for surveillance of tumor recurrence. It can be used in the diagnostic workup of patients with suspicious lesions on imaging studies or patients with symptoms of pancreatic cancer such as obstructive jaundice. However, it is commonly negative in patients with pancreatic cancer and is not a reliable test for the detection of pancreatic cancer even in high-risk symptomatic patients, patients with pancreatitis, patients lacking Lewis antigens, and patients with early-stage cancer [19]. We showed this in our cohort, with stage I and stage II cancers demonstrating a positive CA19-9 in only 57.1% and 44.1%, respectively (Table 3). We now show that a two-gene methylation panel (*ADAMTS1* and/or *BNC1*) demonstrated significant improvement in the detection rate, showing an even higher rate of positivity in stage I and stage II cancers (100% and 94.4%, respectively). However, the addition of CA 19-9 to the two-gene methylation panel did not show any further improvement.

We report a sensitivity and specificity of 87.2% and 95.8% when considering the status of *ADAMTS1*, and 64.1% and 93.7% in the case of *BNC1*. When considering the combination gene panel (*ADAMTS1* and/or *BNC1*), the sensitivity and specificity increased to 97.4% and 91.6% respectively. The high positivity (87.5%) observed in patients with pancreatitis suggests that these markers would be most useful in cases in which chronic pancreatitis can be excluded. Validation investigations in the future should focus on identifying patient populations for whom this panel may not be as predictive, such as those with a history of pancreatitis.

In a previously published study, we had shown that considering both markers results in an overall sensitivity and specificity of 81% and 85% respectively. In this follow-up study, we have continued to optimize our panel, replacing SYBR green with a more specific TaqMan probe results in improvements in both sensitivity (97.4%) and specificity (91.6%). Also, testing our methylation panel on a larger control cohort was helpful in validating the sensitivity and specificity of the two-gene biomarker panel.

Due to its relatively low incidence rate compared to other types of cancer, population-based screening of pancreatic cancer is not reasonable or cost-effective. However, recent publications have shown benefits from screening specific high-risk populations [51–53]. Eight to 10% of patients with a family history of pancreatic cancer or a germline mutation would be candidates for screening along with patients who are long-term smokers [54–59]. Current diagnostic strategies include invasive techniques, offering endoscopic ultrasound screening of selected high-risk groups, or methods that involve extensive use of radiation-based screening [60].

The reduction in lung cancer mortality after using computed tomographic scan (CT scan) for the screening of in-risk population has already been shown by National Lung Screening Trial Research Team [61]. Non-invasive tests which use blood-based biomarkers, such as our suggested one, could be very helpful for the screening of asymptomatic patients, as it reduces the necessity of performing more invasive and expensive tests or biopsies.

Additionally, accurate blood-based biomarkers can be used for following up of previously diagnosed or suspicious pancreatic cancer cases [62, 63]. A recently published study evaluated cancer cell-derived exosomes as a biomarker for pancreatic cancer with promising results [64]. However, this study did not describe the characteristics of the control group, which is essential in determining whether it is representative of the test group from other aspects besides the pancreatic cancer. Furthermore, the previous study included a large number of stage IV patients (21.4%), but a small number of stage I patients (2.6%), which is the stage at which intervention would be most beneficial and therefore should be the focus of a biomarker study [64, 65].

Our study now identifies a two-gene panel with highly promising sensitivity and specificity for detection of earliest stages of pancreatic cancer (sensitivity 97.4% and specificity 91.6%) with an AUC 0.95 (CI 0.91–0.98) as compared to the sensitivity of 55.9% for CA 19-9. The recent FDA approval of DNA methylation-based biomarkers in colorectal cancer highlights the feasibility of our approach as an economical mode of screening high-risk patients.

## Materials and methods

### Clinical samples

The Institutional Review Boards of Johns Hopkins University approved the present study. A total of 142 samples were used for this study (Table 1). Patients with cancer undergoing surgical resection for infiltrating ductal adenocarcinoma of the pancreas were enrolled and blood samples were collected prior to incision (*n* = 39). A control group (*n* = 95) of volunteers was analyzed. The control cohorts enrolled in this study were age-matched patients attending an outpatient clinic for chronic diseases such as diabetes mellitus and hypertension. The exclusion criteria for controls included any history of malignancy and an age of less than 21 years old. In addition, we included eight patients who underwent pancreatic resection due to complicated and long-standing chronic pancreatitis (median years with disease prior to surgery, 84 months) (Table 1). All cohorts were consented and clinical information was obtained as per HIPAA regulations, including pre-operative CA19-9 level for cancer cases (in some cases, the data were not available and this is indicated in respective tables, and available total number were used for calculations). In addition, blood samples were collected and analyzed as per the institutional IRB protocol (NA00033085).

### DNA extraction and bisulfite conversion

The blood specimens were collected using the standard operating procedures, processed using Ficoll-paque (GE health-care), frozen within 2 h of collection, and stored at − 80 °C until utilized. Samples were collected before any surgery or chemotherapeutic treatment. All samples were collected with full institutional IRB approval from the involved centers and informed consent from all individuals who donated blood samples. Plasma obtained after centrifugation was aliquoted into multiple aliquot tubes and stored at − 80 °C. cfDNA was isolated from 2 mL of plasma using novel MOB method as described previously [66, 67]. In brief, MOB is a single-tube processing technique that employs silica-coated magnetic beads for both DNA extraction and bisulfite treatment that results in significant improvements in amplifiable bisulfite-converted DNA over conventional techniques [28, 29, 66].

### Quantitative methylation-specific PCR

We designed our QMSP experiments based on the minimum information for publication of quantitative real-time polymerase chain reaction (PCR) experiment (MIQE) guidelines [68]. Sensitive and specific TaqMan probe-based methods (IDT Inc.) were used for methylation analysis. Primer 3 and Meth Primer programs were used to design all our primers and probes (IDT Inc.). All primer sequences used in the evaluation are listed in Additional file 1: Table S2 and Table S3). Quantitative real-time methylation-specific PCR (QMSP) using StepOnePlus™ Real-Time PCR System (Thermo Scientific) quantified bisulfite-converted cfDNA. The PCR mixture consisted of 2 μl of bisulfite-converted DNA, 200 nM of sense and anti-sense oligonucleotides, 100 nM probe, 100 nM of fluorescein reference dye (Life Technologies), 1.67 mM dNTPs (Thermo Scientific), and 1 μl of Platinum Taq® DNA Polymerase (Invitrogen). The master mix contained 16.6 mM (NH_4_)_2_SO_4_, 67 mM Tris pH 8.8, 6.7 mM MgCl_2_, and 10 mM β-mercaptoethanol in nuclease-free water. Amplification was performed on bisulfite-converted cfDNA with platinum Taq (Invitrogen) in a total volume of 25 μl reaction. Amplification consisted of an initial activation for 10 min at 95 °C, followed by 45 cycles of melting at 95 °C for 30 s, annealing at 60 °C for 30 s, and extension at 72 °C for 30 s. All reactions were performed in triplicates using non-template or water samples as negative controls and CpG methylated Jurkat genomic DNA (IVD) as positive controls (Life Technologies). The quantitative methylation-specific assays were normalized to the levels of the housekeeper gene β-actin. All reactions were performed in triplicate and mean values were considered for statistical analyses.

### Statistical analysis

Stata/MP 14.2 (StataCorp, College Station, Texas) was used for all statistical analysis. Wilcoxon rank-sum test was used to assess the statistical significance of methylation level differences between cancer and control groups. Data was analyzed using ROC analysis using 2^− ΔCt^ values to determine the performance of individual markers which were used for combined detection analysis as described elsewhere [16]. The area under the curve obtained from a ROC curve analysis was used to test the biomarker accuracy. Sensitivity and specificity for each gene was calculated separately and in combination using the cutoff values obtained from the ROC curve.

## Additional file

Additional file 1: **Table S1.** Surgical information. **Table S2.** Oligonucleotides probe sequences. **Table S3.** Oligonucleotides primer and probe design. (DOCX 36 kb)

## Abbreviations

*ADAMTS1*: A disintegrin and metalloproteinase with thrombospondin motifs
AUC: Area under the curve
*BNC1*: Zinc finger protein basonuclin-1
cfDNA: Cell-free DNA
CRC: Colorectal cancer
MOB: Methylation on beads
PCR: Polymerase chain reaction
QMSP: Quantitative real-time methylation-specific PCR
ROC: Receiver operating characteristic curve
