# Supplementary material for: Promoter methylation of ADAMTS1 and BNC1 as potential biomarkers for early detection of pancreatic cancer in blood
Source: Clin Epigenetics. 2019 Apr 5;11:59. doi: 10.1186/s13148-019-0650-0 (PMC6451253; doi:10.1186/s13148-019-0650-0)
Supplement: Supplementary file 1 — Table S1. Surgical information. Table S2. Oligonucleotides probe sequences. Table S3. Oligonucleotides primer and probe design. (DOCX 36 kb) [file 13148_2019_650_MOESM1_ESM.docx]

**Table S1: Surgical information**

|  | Control (n=95) | Cancer (n=39) | Pancreatitis (n=8) |
| --- | --- | --- | --- |
| *Surgical Resection |  |  |  |
| Pancreaticoduodenectomy | - | 27 (69.2%) | 2 (25%) |
| Total pancreatectomy | - | 4 (10.3%) | 4 (50%) |
| Distal pancreatectomy | - | 6 (15.4%) | 0 (0%) |
| Other Surgery | - | 0 (0%) | 2 (25%) |
| Aborted Resection | - | 2 (5.1%) | 0 (0%) |

*Surgical Resection: [PDAC] 2 liver biopsies of stage IV patients, [Pancreatitis] 1 Pancreatic Necrosectomy & 1 Pancreatic Biopsy

| **Primer** | **Sense** | **Antisense** | **Probe** |
| --- | --- | --- | --- |
| *ADAMTS1* | TTAGGGTGCGTTATCGGAC | TAAAACAACGCGAAAATTAATACCTAACG | AGGGGAGAGTTTTGAGTAGAGTGAGTAATATCGTAG |
| *BNC1* | GTTTTTTTTCGGGAGAGGTAAATATCGATAC | CCGACGACCGACG | AGTGTTTTTAAGTTCGGCGGGGGTAG |
| *βactin* | TAGGGAGTATATAGGTTGGGGAAGTT | AACACACAATAACAAACACAAATTCAC | TGTGGGGTGGTGATGGAGGAGGTTTAG |

**Table S2: Oligonucleotides probe sequences**

**Table S3: Oligonucleotides primer and probe design**

**>BNC1 Converted**

TATATTTTAAGACGTTCGTTTCGTATTTTTTCGGGAATGAGGTTTTTGTAGGCGAGGGCGGCGTTGTTTTTTTTTTTCGCGGTAGTGAGATTTCGAGGGCGTTTTAGGGTAGGAGGGGAGGTCGAATTATTTTTTGAGAAGAGCGTTAGAGAATTTTAGAGCGTTTCGTTTTTTTTCGGGAGAGGTAAATATCGATACGTTTGTGTTTTTTATTAATAAGTGTTTTTAAGTTCGGCGGGGGTAGATATTTTCGCGTCGGTCGTCGGCGAGGTTTTCGCGGTTTGCGGGGGTTACGGTTTCGTTTTAGTTGCGTTGATTTAGGGCGTTATTCGGTTTCGGGGCGGGAGGCGGTTTTTCGGGCGGCGAAGTAGCGTTCGCGGCGTGGGGCGATCGCGCGGTGGGCGGAGGGGTAGGGGGAGGGGCGGAGAGGCGTTTTCGGGGCGTAGGGGGCGGGCGTGCGGGTATACGCGGTGCGCGGCGGGGGCGGTTATCGTGTTGCGTAGTTTGGGCGTTTGGGGAGTCGTTTATTTCGTCGGGTCGCGTTTCGACGGTCGGAGCGTGGATGCGGCGGCGTTCGTCGAGTCGGGGCGGACGCGGGGCGGTTCGGGTTCGGGAGACGCGTCGGTAGTTTCGGTATCGTAGCGGTCGTAGGATGGTCGAGGTAAGCGCGGCGTTTTTCGCGGGCGCGGGGATTTTTTGT

**>ADAMTS1 Converted**

AGTTAGCGCGGAGGTTGTTTTTTTTTTTTTTCGAGTTCGTAGCGCGGAGCGCGGTTTAGTATTAACGGAGTCGGGGGCGGCGTTTTTGGGATGGAAAAGGGTTAAAGGGGAGGAGTGGGGTGGGGGTGGGGGTTTTATTGGTTTATTATAAAAGGATCGTTCGGTTGTTCGGTTTTTGTATTCGTTGGAAAGCGGTTTCGAGTTAGGGGTTATTGTAAAGTTAGGGTGCGTTATCGGACGGAGAGGGGAGAGTTTTGAGTAGAGTGAGTAATATCGTAGTTAAGGCGGAGGTCGAAGAGGGGCGTTAGGTATTAATTTTCGCGTTGTTTTAGTTTCGGAGGCGTTTTAGAGCGTTTTTTGTTTTAGTAGAGTTATTTTGTTTGCGTTTGTTTTTTAGTGTTTTTAATTTTGCGTTGGAAGAAAAATTTTTCGCGCGTCGGTAGAATTGTAGCGTTTTTTTTTAGTGATTTCGGGAGTTTCGGTTGTAGTCGGTTTTGCGCGTTTTTTTAACGAATAATAGAAATTGTTAATTTTAATAATTTAGAGTAGGTTAACGAGGTTTTGTTTTTTCGATTCGAATTAAAGGTTTTTCGTTTCGTGCGTTGTTACGAGCGGTGTTTTTTGGGGTTTTAATGTAGCGAGTTGTGTTCGAGGGGTTCGGAAGGCGTAAGTTGGGTAGCGATATGGGGAACGCGGAGCG

Yellow Highlight: probes

Boxes: Sense and Antisense primers
